# Reversed Test Types

**Published:** 1901-11-09

**Authors:** 


### REVERSED TEST TYPES.

A writer in the *British Optical Journal* revives the suggestion that in examining the eye for refraction the test types should be placed behind the patient who should look at them by means of a mirror placed in front of him at half the distance which usually intervenes between the eye and the object. It is a well-known device, but one which is not made use of as often as it might be. Many surgeries and consulting rooms do not permit of the full six metres between the patient and the card, and under such circumstances various allowances have to be made which are apt to detract somewhat from the accuracy of the results; but by placing a good mirror at about three metres from the patient, and making him look at the reflection of the types instead of at the types themselves, the difficulty of the distance is at once got over. There are also several other advantages in this method, among them being the fact that the examiner stands by or behind the patient, and thus is able to change and point to the various tests without crossing the room, while one source of light is sufficient for all purposes. Many a surgeon whose consulting room is only a small one will find that by using a mirror in this way he will save himself much trouble in doing refractions. Reversed letters must be used, and the greatest care must be taken to keep the mirror thoroughly clean, for on this all depends.

